# A nasal airflow oscillation device targeting nasal congestion: a preliminary report

**DOI:** 10.1007/s00405-024-08576-2

**Published:** 2024-03-04

**Authors:** Jim Bartley, Robin Hankin

**Affiliations:** 1 Department of Otolaryngology - Head and Neck Surgery, Manukau Superclinic, 901 Great South Road, Manukau City Centre, Auckland, 2104 New Zealand; 2 Computer and Mathematical Sciences, University of Stirling, Stirling, FK9 4LA Scotland, UK

**Keywords:** Allergic rhinitis, Rhinitis, Sinusitis, Therapeutics, Vibration, Nitric oxide

## Abstract

**Purpose:**

Upper respiratory tract complaints are common in the general population. A safe, non-pharmacologic treatment would be an attractive option for many patients either as an alternative to existing therapies, or as a complementary therapy. This study assessed the acceptability, safety and possible efficacy of a nasal airflow oscillation device in a group of people suffering chronic nasal congestion.

**Methods:**

Subjects with a known history of nasal congestion, but without fixed anatomical obstruction, participated in a prospective clinical study. Efficacy was assessed using peak nasal inspiratory flow (NPIF) and a 10-point visual analogue scale (VAS) administered before and after the oscillation device had been worn for twenty minutes.

**Results:**

Twenty-one subjects (mean age 37 years; 43% female) were enrolled in the study. After treatment with the small nasal airflow oscillation device for twenty minutes, average NPIF increased significantly from 84.8 L/minute to 99.0 L/minute (*p* < 0.05). There was a corresponding significant reduction in the VAS score for nasal congestion (*p* < 0.05). Similar significant improvements were also seen for the immediate sensation of nasal drainage, sinonasal pressure and overall sinonasal symptoms (*p* < 0.05). There was no change in the sense of smell (*p* = 0.37). Subjects rated ease of use highly; average = 9.1 (Range 7–10).

**Conclusion:**

Treatment of nasal congestion with the nasal airflow oscillation device was found to result in significant improvement in NPIF after twenty minutes of use. Initial patient-reported outcomes improved significantly, and the treatment was safe and highly acceptable.

**Trial registration:**

Public clinical trial registration: Universal Trial Number (U1111-1259-0704). Australian New Zealand clinical trials registration: ACTRN12623001307695.

## Introduction

Nasal congestion affects roughly 20% of the worldwide population [1]. Nasal congestion can be associated with allergic rhinitis and chronic rhinosinusitis (CRS). Depending on the symptoms and diagnostic criteria, allergic rhinitis affects up to 10–20% of the population [2]; while some overlap is present, CRS affects approximately 5–12% of the population [3].

The internal nasal respiratory surfaces are lined by pseudostratified, ciliated, columnar epithelium. A thin, liquid layer lines the upper and lower respiratory tract airway epithelium. This thin, liquid layer is comprised of a pericilial, or sol layer, surrounding the cilia and overlaying the airway mucosa, and an overlying mucus gel blanket facing the airway lumen [4]. The airway surface liquid layer hydration and mucociliary clearance are influenced by the airflow-induced shear stress and transepithelial pressure gradients generated by tidal breathing [5]. In cystic fibrosis sputum samples, oscillatory therapy can break down high-molecular-weight DNA and decrease mucus viscoelasticity [6]. Airflow oscillation may reduce mucus viscoelasticity and enhance mucus clearance [7].

Nitric oxide (NO) is produced in the ciliated epithelial cells The paranasal sinuses are a rich endogenous NO source [8, 9]. Intranasal NO increases nasal blood flow and mucociliary activity [10, 11]. Humming with the lips closed increases nasal NO levels [12]. A randomised controlled study involving sixty people with CRS found that regular humming, as assessed by the Sino-Nasal Outcome Test- 22 (SNOT-22) score, is beneficial in CRS management [13]. In a pilot study, a SinuSonic device, which combines acoustic vibration with oscillating expiratory pressure at approximately 128 Hz, improved visual analogue scale (VAS) symptoms of nasal congestion [14]. After 2 and 5 weeks of twice-daily treatments, the SinuSonic device improved peak nasal inspiratory flow (NPIF), Total Nasal Symptom (TNS), Nasal Obstruction and Septoplasty Evaluation (NOSE), and SNOT- 22 scores [15].

**Fig. 1:**
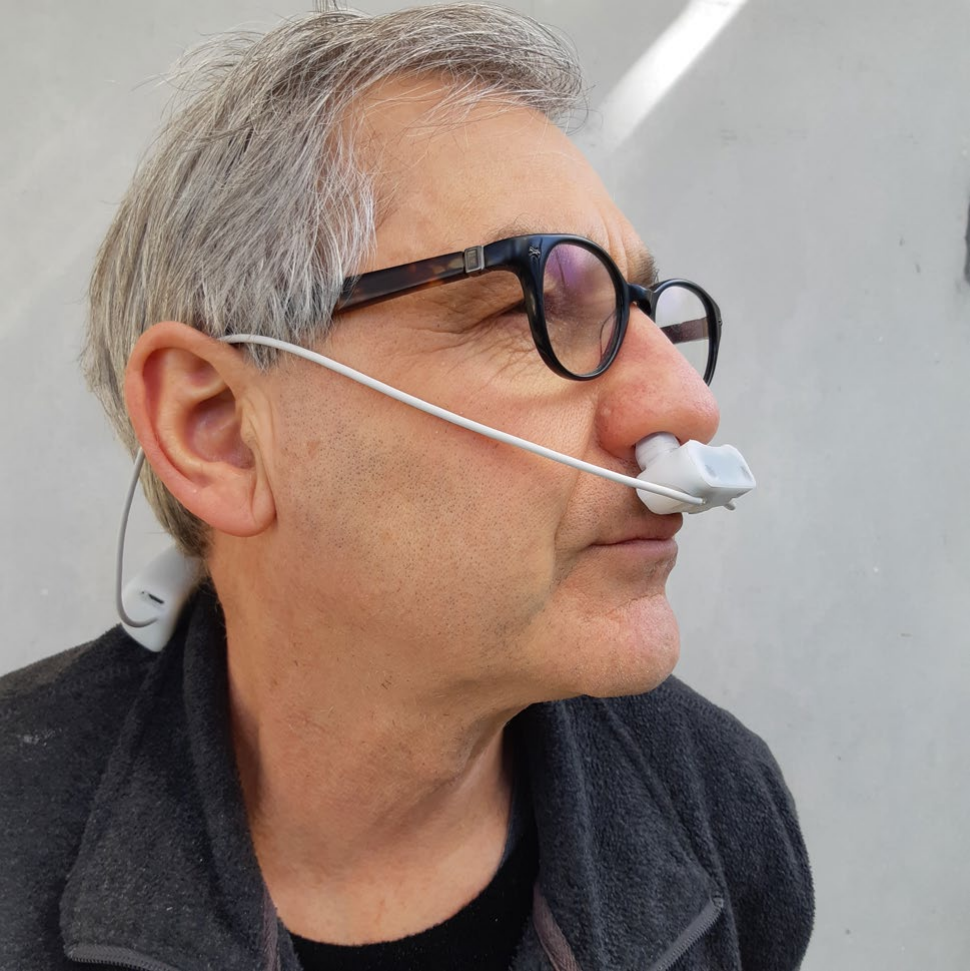
Nasal airflow oscillation device

Based on the above observations, a small nasal airflow oscillation device that plugs into the nose (Fig. 1) to create nasal pressure oscillations at 130 Hz was developed. Unlike unassisted humming and the SinusSonic device, which can only be done during exhalation, this technology also creates pressure oscillations during inhalation, enabling endogenous NO to be drawn into the airways, rather than mainly exhaled and lost. The purpose of this study was to assess the acceptability, safety, and possible efficacy of this small nasal airflow oscillation device in subjects suffering nasal congestion.

## Subjects and methods

The study was approved by the Health and Disability Ethics Committee (21/CEN/99), a Universal Trial Number was obtained (U1111-1259-0704), and the trial was registered with the Australian New Zealand Clinic Trials Registry (ACTRN12623001307695). Subjects with a known history of persistent nasal congestion, were approached, and invited to participate. Inclusion criteria were adults aged from 18 to 80 years with a history of chronic nasal congestion for more than one year. Exclusion criteria included cigarette smoking, a fixed structural cause of nasal congestion (moderate or severe septal deviation), moderate or severe nasal valve collapse, Grade 2–4 polyps, recent upper respiratory illness, nasal decongestant use within the last week, nasal crusting or ulceration on rhinoscopy, a history of severe nose bleeding within the last 3 months, anticoagulant use, known pregnancy, allergic sensitivity to silicone or any other component of device, and inability to read and understand English. All subjects provided written informed consent in accordance with the Health and Disability Ethics Committee (21/CEN/99), a Universal Trial Number was obtained (U1111-1259–0704), and the trial was registered with the Australian New Zealand Clinic Trials Registry (ACTRN12623001307695).

All subjects were evaluated at baseline by an otolaryngologist (JB), who assessed the subjects’ past medical history, and who performed rhinoscopy to screen for exclusion criteria. Those patients with prior positive allergen-specific immunoglobulin E (IgE) testing or positive skin testing for environmental allergens were considered to have allergic rhinitis. Patient demographics and the use of medications for rhinitis symptoms (nasal steroid sprays, nasal antihistamines, oral antihistamines, mucolytics and leukotriene modifiers) were recorded (Table 1).

**Table 1:** Baseline characteristics of the study cohort

Characteristic	Value
Age (years), mean (range)	37 (20–74)
Demographics	
Sex, *n* (%)	
Male	12 (57%)
Female	9 (43%)
Ethnicity, *n* (%)	
European	11 (52%)
Middle Eastern	1 (5%)
Māori	3 (14%)
Asian	4 (19%)
Pacific Islander	2 (7%)
Comorbidities, *n* (%)	
Allergic rhinitis	6 (29%)
Non-allergic rhinitis	14 (67%)
Chronic rhinosinusitis	1 (5%)
Current medication use, *n* (%)	
Nasal steroid spray	1 (5%)
Oral antihistamine	1 (5%)
Rhinoscopy findings, *n* (%)	
Nil of note	19 (90%)
Septal deviation (mild)	2 (10%)
Nasal valve collapse	0
Ulceration	0
Crusting	0

### Baseline assessments

After initial rhinoscopy, baseline nasal peak inspiratory flow (NPIF) was performed on each subject [16]. The otolaryngologist (JB) trained each patient to perform NPIF. After an initial training run, three runs were then performed, averaged, and recorded in litres/minute (L/ min). Subjects were then asked to rate individual nasal symptoms including overall sinonasal symptoms using a 10-point visual analogue scale (VAS), with higher scores generally representing greater symptom burden. However, a higher VAS smell score represented a higher sense of smell. The principal investigator discussed the prototype status of the current device, and the experimental nature before asking the subject to trial it. The subject was then asked to rate their initial experience of breathing at rest on a 1–10 scale (10 was easiest) while wearing the device. The subject was then asked to wear the device for 20 min. The device was then removed. NPIF was then repeated on three occasions, averaged, and recorded in L/min. The otolaryngologist then repeated the rhinoscopy. Patients then re-rated their nasal symptoms using the 10-point VAS.

### Statistical analysis

Data were analysed for within subject differences using basic statistical tests of relationship and difference using two-sided *t* tests. A value of *p* < 0.05 was considered statistically significant.

## Results

A total of 21 patients were enrolled in this first phase, with an average age of 37 years (range 20–74 years). Women accounted for 43% of the cohort, and with a racial make-up like New Zealand overall (Table 1). Six patients were classified as having allergic rhinitis. Two patients had a mild septal deviation, and no patient had nasal valve collapse. One patient with mild CRS was included as that regular humming, as assessed by the Sino-Nasal Outcome Test- 22 (SNOT-22) score, is beneficial in CRS management (Table 1). After treatment with the small nasal breathing oscillation device for twenty minutes average NPIF significantly increased (*p* < 0.05) from 84.8 L/min to 99.0 L/min (Table 2). There was a corresponding significant reduction (*p* < 0.05) in VAS score for nasal congestion (Table 2). Similar significant improvements (*p* < 0.05) were also seen for the immediate sensation of nasal drainage, sinonasal pressure and overall sinonasal symptoms. There was no significant change in the sense of smell (*p* = 0.37) after short-term use. No patient experienced bleeding, and no ulceration or bleeding was identified on repeat rhinoscopy. Patients rated ease of use highly; average = 9.1 (range 7–10).

**Table 2:** Post-treatment (20 min) assessments

Assessment	Baseline (mean ± SD)	20 Minutes (mean ± SD)	*p* value
Objective assessment (*N* = 21)			
NPIF	84.8 ± 39.6	99.0 ± 40.5	< 0.05
Nasal symptoms VAS (*N* = 21)			
Congestion	4.7 ± 1.8	3.5 ± 1.7	< 0.05
Drainage	3.2 ± 2.2	2.4 ± 2.1	< 0.05
Pressure	4.5 ± 1.7	2.6 ± 1.8	< 0.05
Smell	5.2 ± 2.4	4.9 ± 2.9	*p* = 0.37
Overall	4.9 ± 1.7	3.5 ± 2.1	< 0.05

*VAS* visual analogue scale

## Discussion

Nasal congestion impacts roughly 20% of the worldwide population [1]. Patients with chronic rhinitis symptoms report significant reductions in quality-of-life measures related to physical, mental, and social functioning [3]. A variety of pharmacologic options, including topical nasal steroid sprays and oral antihistamines, prolonged antibiotic, or oral Prednisone use, as well as surgery are used to manage nasal congestion associated with allergic rhinitis and CRS [1, 17]. One patient with mild CRS was included as that regular humming, as assessed by the Sino-Nasal Outcome Test- 22 (SNOT-22) score, is beneficial in CRS management [13]. A safe non-pharmacologic treatment would be an attractive option for many patients either as an alternative to existing therapies, or as a complementary therapy. The role of vibrational technology to manage sinonasal pathology is receiving increasing attention [18]. Unlike the SinuSonic device, which combines acoustic vibration with oscillating expiratory pressure at approximately 128 Hz [12, 13], this technology also creates pressure oscillations at 130 Hz during inhalation, enabling endogenous NO to be drawn into the airways, rather than mainly exhaled and lost.

This study provides initial data on the safety and acceptability, and initial efficacy of a nasal airflow oscillation device in a group of patients suffering chronic nasal congestion. After twenty minutes use, no adverse effects were reported or identified, and patients rated the ease of use highly. This suggests that the use of a small nasal breathing oscillation device carries minimal risk in appropriately selected patients.

Objective changes in NPIF were seen after twenty minutes. The subjects had a baseline NPIF of 84.8 L/min. The mean value of subjects with no nasal obstruction is 138.4 L/min, and the mean value of nasally obstructed populations is 97.5 L/min [19]. Thus, our patients had a significantly impaired baseline NPIF consistent with their complaints of nasal congestion. Prior reports use both absolute improvement and percentage of baseline improvement to gauge success. Our patients experienced an absolute improvement of 14.2 L/min (16.7% improvement over baseline). A previously reported mean clinically important difference (MCID) for NPIF of 20% of baseline has been reported [20]. The MCID of 20% of baseline was achieved in nine patients.

Nasal symptoms are primarily a quality-of-life issue, and therefore patient-reported metrics are important. A VAS scale of nasal symptoms was used, as the TNS score, NOSE scale and SNOT-22 evaluate nasal symptoms over a longer time period. In this study, significant improvements were seen in immediate sensation of nasal congestion, nasal drainage, sinonasal pressure and overall sinonasal symptoms.

As an initial clinical study, this study focused on establishing the acceptability and safety, and exploring the possible clinical efficacy of a nasal airflow oscillation device. Objective (NPIF) and patient-reported outcome metrics were used. More commonly used outcome measured such as TSN, NOSE and SNOT-22 scores were not used because of the brevity of the trial. The placebo effect cannot be excluded; however, patients did report that their sense of smell remained largely unchanged. The inclusion of NPIF as an objective measure demonstrated that changes were not just related to patient perceived metrics. Blinding patients and administering a sham would be difficult considering patients can feel the acoustic vibration. Although NO is the most widely studied molecule in this regard, precise mechanisms remain an area for future study. Additional investigations into how long immediate symptomatic improvement occurs, the optimal frequency of acoustic vibrations and duration of treatment will need further evaluation.

## Conclusions

Treatment of nasal congestion with acoustic vibration was found to result in significant improvement in NPIF after twenty minutes of use. Patient-reported outcomes were significantly improved, and the treatment was safe and highly acceptable.
